# A high-quality genome of an undescribed *Flavobacterium* species uncovered using Q20+ Nanopore chemistry

**DOI:** 10.1128/mra.00716-23

**Published:** 2023-12-01

**Authors:** Cyrill Hofer, Denise Wälchli, Lucas Serra Moncadas, Angel Rain-Franco, Adrian-Stefan Andrei

**Affiliations:** 1 Limnological Station, Department of Plant and Microbial Biology, University of Zurich, Kilchberg, Switzerland; 2 Faculty of Science, University of Zurich, Zurich, Switzerland; Indiana University, Bloomington, Bloomington, Indiana, USA

**Keywords:** genomics, DNA sequencing, Nanopore, *Flavobacterium*

## Abstract

Herein, we document the complete genome of the *Flavobacterium* strain ZE23DGlu08, isolated from Lake Zurich, Switzerland. The circular genome was assembled using long-read Nanopore data (coverage: 226×) with the Q20+ chemistry. The described strain displays a genome size of ~3.9 Mbp with a GC content of 34%.

## ANNOUNCEMENT

While some representatives of the genus *Flavobacterium* are known as severe fish pathogens (1), the vast majority exhibit a free-living lifestyle in wet soils, rivers, lakes, and oceans (2). Primarily, Flavobacteria are aerobic and chemo-organotrophic organisms specialized to degrade high-molecular-weight-dissolved organic compounds, thereby contributing to the carbon cycle of aquatic ecosystems (3).

The strain was isolated from the surface water of Lake Zurich (47°18′N, 8°34′E, Switzerland) and cultivated in agar plates (15 g/L) amended with artificial lake water with cellobiose as carbon substrate (1 mM final concentration), following a published protocol (4). After 21 days of cultivation, colonies were purified through three sequential streaking cycles and then cryopreserved (5). The isolated strain was reactivated in artificial lake water (4), supplemented with glucose (100 µM) for 1 week, and grown in commercial LB medium (Difco, 240210) for 2 days. DNA extraction was performed using the Quick-DNA HMW MagBead Kit (Zymo Research) and cleaned with Agencourt AMPure XP beads (Beckman Coulter) according to the manufacturer’s guidelines. Library preparation was done using the Native Barcoding Kit 24 V14 (SQK-NBD114-24, Oxford Nanopore) by processing 1 µg of pure (A260/280 = 1.824, A260/230 = 2.252) high molecular weight DNA (~50 kb, GeneRuler High Range DNA Ladder—Thermo Scientific). Without additional shearing, 154.5 ng was loaded on a FLO-MIN114 R10.4.1 flow cell and sequenced on the minION Mk1B (Oxford Nanopore). After 72 h, the sequencing yielded 1,272,573 reads passing the quality score of Q10 (mean quality: Q14.2; N50: 6,610 bp) before further filtration using chopper [v.0.2.0 (6)] to retain high-quality reads only (≥Q17, ≥1,000 bp). The raw data were basecalled using guppy [v.6.4.6 (7); super accurate basecalling, 400 bps]. The obtained file (177,273 reads; mean quality: Q18.1; N50: 6,819 bp) was *de novo* assembled using the Flye assembler [v.2.9.2-b1786 (8)] by specifying the corrected Nanopore data option, supported by the high read quality with less than 3% average error (flye --nano-hq –meta). One circular contig (identified and trimmed by Flye) was recovered with a length of 3,893,827 bp and a GC content of 34% (99.29% completeness, 0.24% contamination). The quality of the assembled sequence was assessed by CheckM [v.1.2.2 (9)], before whole-genome taxonomic classification with GTDB-Tk [v.2.2.6 (10)] against the Genome Taxonomy Database 214.1 (11). The 16S rRNA genes were extracted using barrnap [v.0.9 (12)] and classified with SILVA 138.1 SSU (13) and NCBI (14). Gene prediction was performed using Prokka [v.1.12 (15)] as well as NCBI’s PGAP [v.6.5 (16)] pipeline, followed by annotation, harnessing the PFAM (17), KEGG (18), MetaCyc (19), and NCBI (14) databases. Default parameters were used except where otherwise noted.

The isolated strain was classified as an uncultured *Flavobacterium* species displaying 98.15% (coverage: 90%) whole-genome similarity with a genome recovered from freshwater sediments (i.e., GCF_002754315.1). The closest described species was found to be *Flavobacterium franklandianum* [NCBI (14): MK346164.1] sharing a 98.55% 16S rRNA identity (coverage: 100%), despite the low-genome similarity of 77.84% (coverage: 42.61%). The genome-resolved functional annotation of the 3,355 genes revealed four intact rRNA operons, such as complete amino acid biosynthesis, glycolysis and glycogenesis, the presence of the TCA cycle, and oxidative phosphorylation. In conclusion, this genome belongs to a copiotrophic *Flavobacterium* species that exhibits a non-motile, free-living, aerobic, and heterotrophic lifestyle.

## Data Availability

The sequence data and additional information are available at NCBI, BioProject PRJNA991734 (Accession: CP130045, BioSample: SAMN36315993, SRA: SRR25177534).
